# I*GF2R* circular RNA hsa_circ_0131235 expression in the middle temporal cortex is associated with AD pathology

**DOI:** 10.1002/brb3.2048

**Published:** 2021-03-11

**Authors:** Inès M. Bigarré, Bianca A. Trombetta, Yan‐jun Guo, Steven E. Arnold, Becky C. Carlyle

**Affiliations:** ^1^ Toulouse Purpan Medical School Toulouse France; ^2^ Department of Neurology Massachusetts General Hospital Boston MA USA; ^3^ Department of Neurology Beijing Friendship Hospital Capital Medical University Beijing China; ^4^ Harvard Medical School Boston MA USA

**Keywords:** Alzheimer's disease, circular RNA, I*GF2R*, middle temporal cortex

## Abstract

**Objective:**

To identify circular RNAs as candidates for differential expression in the middle temporal (MT) cortex in a well‐characterized cohort with contrasting Alzheimer disease (AD) pathology and cognition. Top screen candidates were assessed for proof of circularity and then quantified by qPCR in a larger number of samples.

**Methods:**

An initial RNA sequencing screen was performed on *n* = 20 frozen human tissue samples. Filters were applied to select candidate circular RNAs for further investigation. Frozen human tissue samples were selected for global AD pathology burden and global cognition scores (*n* = 100). Linear and divergent primers were used to assess circularity using RNaseR digestion. RT‐qPCR was performed to quantify relative hsa_circ_0131235 abundance.

**Results:**

Eleven circular RNAs were selected for further investigation. Four candidates produced circular RNA primers with appropriate efficiencies for qPCR. RNaseR treatment and analysis by both basic PCR and qPCR confirmed hsa_circ_0131235 circularity. There was a significant main effect of AD pathology on hsa_circ_0131235 expression.

**Conclusions:**

Elevated hsa_circ_0131235 expression in the MT cortex was significantly associated with AD pathology.

## Graphical abstract

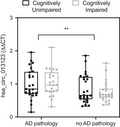

100). Linear and divergent primers were used to assess circularity using RNaseR digestion. RT‐qPCR was performed to quantify relative hsa_circ_0131235 abundance. RNaseR treatment confirmed hsa_circ_0131235 circularity. There was a significant main effect of AD pathology on hsa_circ_0131235 expression. Elevated hsa_circ_0131235 expression in the MT cortex was significantly associated with AD pathology

## 1 INTRODUCTION

RNAs (circRNAs) are noncoding RNAs generated by back splicing, which creates an identifiable covalent junction between the exon 5′ and 3′ ends (Perera et al., 2018). CircRNAs are highly abundant in the mammalian brain (Rybak‐Wolf et al., 2015), more stable than linear RNAs, enriched in synaptoneurosomes, and upregulated during neural differentiation (You et al., 2015). Although little is currently known about their specific roles in the nervous system, circRNA expression is distinct from linear isoforms of the same gene, suggesting an independent function. A leading hypothesis is that they may act as a “sponge” to regulate microRNA expression in the central nervous system (CNS; Hansen et al., 2013; Piwecka et al., 2017). For example, CDR1 circRNA has been shown to negatively regulate miR‐7 microRNA, which plays a role in sensory motor gating and synaptic transmission (Piwecka et al., 2017). Due to their high stability, circRNAs may also serve as synaptic tags to maintain molecular memory (Rybak‐Wolf et al., 2015). A number of circRNAs have been previously associated with AD pathology and may explain more of the variation in clinical dementia rating of AD cases than ApoE4 risk gene load (Dube et al., 2019).

In order to identify circRNAs that may be involved in AD pathophysiology, we performed a small RNA sequencing screen of potential circRNAs in frozen tissue from the human middle temporal (MT) cortex. Using a series of filters, we selected 11 candidate circRNAs for follow‐up. Following assessment of circularity, 1 target, IGFR2, was quantified by RT‐qPCR in a larger sample set. This cohort included well‐matched individuals with contrasting presentations of AD pathology and cognition, to allow for somewhat independent evaluation of circRNA association with cognition or pathology. IGFR2 hsa_circ_0131235 was established as clearly circular, and expression was found to be associated with AD pathology.

## 2 METHODS

### 2.1 Human tissue

Frozen human tissue was used according to human research protocols as approved by the Partners Institutional Review Board. 100 cases from the Rush Religious Orders Study (ROS) and Memory and Aging Project (MAP) cohorts (Bennett et al., 2013) were selected according to global AD pathology burden and the presence of cognitive impairment. Subjects were classified into four contrasting groups according to two variables. The first variable was Braak Score, with those individuals with a Braak score of 4 or below deemed low AD pathology burden, and those with a Braak score of 5 or 6 deemed high AD pathology burden. The second variable was clinical consensus as to the presence of cognitive impairment at the point of death.

### 2.2 RNA sequencing

Exploratory RNA sequencing was performed on frozen human MT tissue (*n* = 20, 5 samples per diagnostic group) to identify candidate circRNA targets. Reads were aligned to the hg19 genome using STAR aligner, and read counts were normalized using DESeq2 (Love et al., 2014). High confidence (KNIFE score > 0.9) circRNAs were identified by the presence of head‐to‐tail splice junctions using the KNIFE algorithm (Hansen, 2018). The table of circRNA counts per sample (Table S1) was filtered as follows: (a) CircRNA detected in at least 4 out of 20 samples, (b) circRNA detected in at least two diagnostic groups, and (c) the sum of reads in at least one group should be >10.

### 2.3 Primer design

Primers for reference genes (RGs) and target genes were designed using Primer‐BLAST (NCBI). Eight RGs were evaluated based on published stability in human brain tissue (Rydbirk et al., 2016). Primers were subjected to a 10‐fold dilution series and efficiency calculated using the formula: (10^−1/slope)−1^). *UBE2D2* and *RPL13* demonstrated good efficiency in these samples and were used as reference genes (Table 1).

**TABLE 1:** Gene targets and associated control and divergent primers. Primer sequences, efficiencies, and predicted product size listed for each primer pair

Gene name	Circbase reference	Control primers	Divergent primers	Control efficiency	Divergent efficiency	Control product (bp)	Divergent product (bp)
chr12|FGD6:95602618|FGD6:95605043|dup|−	hsa_circ_0099549	F: 5′‐GCACTGAAGAACCGGGGAAT−3′, R: 5′‐GCTGCTTAGGCAGGCTCATA−3′	F: 5′‐GAGGACGCTGATGCAAATGT−3′, R: 5′‐TGGGGCTATTGCTGGTTTCAT−3′	0.9088	1.0636	366 bp	238 bp
chr4|TBCK:107092251|TBCK:107133992|rev|−	hsa_circ_0007540	F: 5′‐TCCAGATCTCTATGCCATCCCT−3′, R: 5′‐AGGTTGAGCATGCTGTCTGT−3′	F: 5′‐GGCAGAAGTTCGGCACCTTA−3′, R: 5′‐ACCAAGGGATGGCATAGAGA−3′	1.1629	1.0573	295 bp	165 bp
chr6|I*GF2R*:160469575|I*GF2R*:160467529|rev|+	hsa_circ_0131235	F: 5′‐GAGGCGGCACACCCTATAAC−3′ , R: 5′‐CTGGTGTACCACCGGAAGTT−3′	F: 5′‐AGCAGTACGACCTCTCCAGT−3′, R: 5′‐ACCGGGCCACACACATTTAT−3′	0.9566	1.1879	142 bp	285 bp
chr6|MAP3K4:161471011|MAP3K4:161469647|dup|+	hsa_circ_0078619	F: 5′‐TATGGGAGCTTCGCCTTTGT−3′ , R: 5′‐CTGGCCAGCCAATGTCTGAT−3′	F: 5′‐AGGGCACGTATAGCATTGGT−3′, R: 5′‐GTCCTAGAAGTCTGGCGTGC−3′	0.9817	1.2972	364 bp	346 bp
chr11|GAS2:22777499|GAS2:22696395|rev|+	hsa_circ_0095626	F: 5′‐GCCTTGCTCTGTCAACTTGC−3′ , R: 5′‐GACACGTTTCATCCACCCCT−3′	F: 5′‐GTGGAGCCTCCTGGTTTGAT−3′, R: 5′‐TAGCTTCATGTCTGCTGGCT−3′	1.0309	1.2386	196 bp	357 bp
chr7|FAM126A:22999874|FAM126A:23030758|rev|−	hsa_circ_0008951	F: 5′‐TGAACCTTCCAGCATTGGGT−3′ , R: 5′‐GCTGTCAGCATCTCCCTTTGA−3′	F: 5′‐GAGCCATTGCTGGTTGCTAAT−3′, R: 5′‐CTCACTTTGTGGCTCCTGGAT−3′	1.0080	0.8716	111 bp	353 bp
chr9|RABGAP1:125782738|RABGAP1:125719289|rev|+	hsa_circ_0137854	F: 5′‐AGAATACACGTCTTCCGGTGT−3′ , R: 5′‐TCCTGGACTAAGGAGAAGACCA−3′	F: 5′‐AGAAACGGTGTCCCTGAAGC−3′, R: 5′‐TGGTGTCTCATCTCCTTGCC−3′	0.9646	1.2039	333 bp	262 bp
chrX|CDKL5:18528974|CDKL5:18525054|rev|+	hsa_circ_0089980	F: 5′‐TCCCACCAACCAGTGAGAATTT−3′ , R: 5′‐CTCCTCTTTTGATGCAAGCCAC−3′	F: 5′‐GATCCTTGGGGTTGTAGGTGA−3′, R: 5′‐ATTCTCACTGGTTGGTGGGAAC−3′	1.1319	1.8453	110 bp	121 bp
chr2|CLASP1:122363276|CLASP1:122363756|dup|−	hsa_circ_0007052	F: 5′‐CAGCGAACAAAGAGGCAACC−3′ , R: 5′‐CAACCTGCAATCGTTTCCCC−3′	F: 5′‐GATTGCAGGTTGGCCAAGAAC−3′, R: 5′‐GAGTGAGTGGCAGAGTGATGT−3′	0.9948	1.9461	166 bp	201 bp
chr2|PGAP1:197777605|PGAP1:197784874|rev|−	hsa_circ_0003394	F: 5′‐CTCCCTTTGACGGGTATTCCA−3′ , R: 5′‐TGCAACAAGGCCACCCATAG−3′	F: 5′‐ACAAGCCACACCTCATGTTG−3′, R: 5′‐ACTCATATGCGGGATAGCGTTT−3′	1.0468	1.6296	300 bp	102 bp
chr6|SOBP:107827631|SOBP:107824860|rev|+	hsa_circ_0001633 (alias hsa_circ_000737)	F: 5′‐GAGCTACCCTACAGATGGGGA−3′ , R: 5′‐TGCCATGATCCTTTGACCCT−3′	F: 5′‐AGGGTCAAAGGATCATGGCA−3′, R: 5′‐CCATACCAGCCAAGGAGTTCA−3′	1.1459	1.7760	182 bp	118 bp
UBE2D2	NA	F: 5′‐AGAGAATCCACAAGGAATTGAATGA−3′, R: 5′‐ACTCCACCCTGATAGGGACT−3′	NA	1.1056	NA	136 bp	NA
RPL13	NA	F: 5′‐CTTTCCGCTCGGCTGTTTTC−3′, R: 5′‐GCCTTACGTCTGCGGATCTT−3′	NA	0.9393	NA	164 bp	NA

### 2.4 RNA digestion

Total RNA was extracted from frozen tissue (50mg) using miRNeasy (QIAGEN). RNaseR digestion was used to confirm RNA circularity exactly as described (Panda & Gorospe, 2018). This experiment was independently replicated 4 times.

### 2.5 cDNA synthesis

cDNA was synthesized using Maxima Reverse Transcriptase (Thermo Fisher). 12 µl RNaseR‐treated or vehicle‐treated RNA was used to assess circularity, and 5 µg Total RNA was used for quantification.

### 2.6 Agarose gel electrophoresis

cDNA was used in basic PCR reactions for visualization on an agarose gel. Reactions were performed using PCR Master Mix (Thermo Fisher) on a C1000 Touch Thermal Cycler (Bio‐rad) system using standard conditions (30 s extension, 40 cycles), 1 μmol/L primers, and 1 μL of cDNA. PCR products were loaded onto a 2% agarose gel containing SybrGold.

### 2.7 RT‐qPCR

cDNA was used for reverse transcriptase quantitative PCR (RT‐qPCR). Reactions were performed in triplicate using RT^2^ SYBR Green MasterMix (QIAGEN) on a CFX96 (Bio‐rad) system using standard cycling conditions, (10 min at 95°C followed by 40 cycles of 15 s at 95°C, 90 s at 60°C), 0.4 µmol/L primers, and 1 µl cDNA. Control and divergent target gene primers were used for circularity confirmation. Only divergent target gene primers were used for differential expression quantification.

### 2.8 Statistical analysis

Statistical analyses were performed using GraphPad Prism (v7). RT‐qPCR data were analyzed using efficiency adjusted ΔΔCT ([2*efficiency]^ΔCT^) and normalized to RG expression. Independent *t* tests were used to analyze cohort demographics and RNaseR‐treated RNA enrichment. Two‐way ANOVA was performed to examine the effect of pathology and cognition on circRNA expression.

## 3 RESULTS

### 3.1 RNA sequencing

RNA sequencing was performed on RNA from 20 samples from the human MT cortex (Table S2), to a read depth of between 35 million to 52 millions reads per sample in all but one sample. 9,670 circRNAs were identified by the KNIFE algorithm as high confidence circRNAs in at least one sample, but the vast majority were identified in fewer than 3 samples, and at very low read counts (Table S1). The table of circRNA counts per sample was filtered to a small number of potential targets using the following criteria: (a) CircRNA should be detected in at least 4 out of 20 samples, (b) circRNA should be detected in at least two diagnostic groups, and (c) the sum of reads in at least one group should be >10. Applying these filters reduced candidate circRNAs down to 161 potential targets. The remaining 161 circRNAs were subjected to exploratory *t* tests for the cognition variable and the AD pathology variable. A shortlist of eleven targets was selected for quantification by qPCR by *p*‐value, prior annotation in circBase, and biological interest of the parent gene to Alzheimer's Disease pathophysiology: MAP3K4: hsa_circ_0078619, TBCK: hsa_circ_0007540, FGD6: hsa_circ_0099549, I*GF2R*: hsa_circ_0131235, SOBP: hsa_circ_0001633, PGAP1: hsa_circ_0003394, RABGAP1: hsa_circ_0137854, FAM126A: hsa_circ_0008951, GAS2: hsa_circ_0095626, CLASP1: hsa_circ_0007052, and CDKL5: hsa_circ_0089980.

### 3.2 PCR primer testing

To ensure accurate quantification of linear and circRNA targets, efficiency testing was first performed on each primer pair. This is particularly important for the circRNAs, where the primer must cross the head‐to‐tail splice junction and thus there is little choice in location for primer design. Primer pairs with efficiencies of <0.8 or >1.2 were eliminated from further experiments (Table 1). I*GF2R*: hsa_circ_0131235, FAM126A: hsa_circ_0008951, FGD6: hsa_circ_0099549, and TBCK: hsa_circ_0007540 were taken forwards for confirmation of circularity.

### 3.3 Circular RNA validation

**FIGURE 1:**
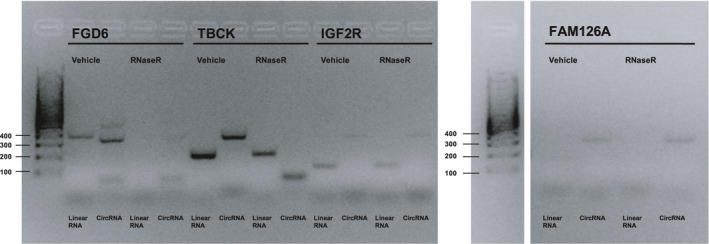
PCR amplification of gene targets that met primer pair efficiency criteria. Expected product sizes for control and divergent primer pairs, respectively: FGD6, 366 bp, 238 bp; TBCK, 295 bp, 165 bp; I*GF2R*, 142 bp, 285 bp; FAM126A, 111 bp, 353 bp

**FIGURE 2:**
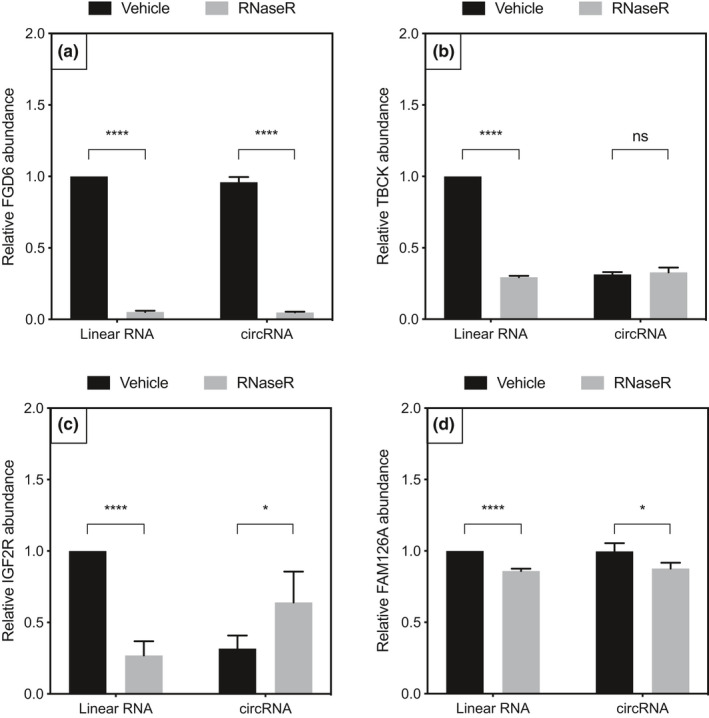
qPCR quantification of RNase‐treated gene targets 2A) FGD6, 2B) TBCK, 2C) I*GF2R*, and 2D) FAM126A. RNaseR‐treated linear RNA expression was significantly decreased compared to vehicle for all four gene targets. RNaseR‐treated circRNA expression was enriched compared with vehicle (*p* < .05) for TBCK and I*GF2R*. *p*‐value significance codes: ****p < .0001, ***p < .001, **p < .01, *p < .05

To confirm RNA circularity was not an artifact of cDNA synthesis, circularity was established using RNaseR treatment (Panda & Gorospe, 2018). RNaseR should digest linear mRNAs while leaving most circRNAs intact. RNA was amplified by PCR and run on a gel for visual inspection. I*GF2R* hsa_circ_0131235 and *FAM126A* hsa_circ_0008951 showed visual patterns of banding likely consistent with true RNA circularity (Figure 1). TBCK: hsa_circ_0007540 produced a smaller product on RNaseR digestion that was not consistent with circularity for the appropriate target. All four targets were further quantified by qPCR post‐RNase treatment (Figure 2). For all four linear mRNAs, control primer PCR product was decreased by RNaseR treatment. I*GF2R* hsa_circ_0131235 and TBCK: hsa_circ_0007540 were the only circular RNAs to show stable products upon RNase treatment. Only I*GF2R* hsa_circ_0131235 was taken forwards to quantification in the larger sample set by qPCR, given the band of incorrect size seen in the RNase digested TBCK: hsa_circ_0007540 lane.

### 3.4 Demographics of validation cohort

**FIGURE 3:**
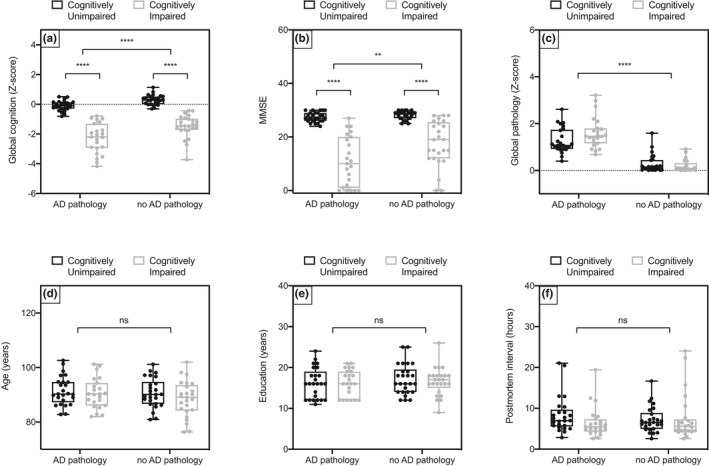
Plots to show the individual demographics of all subjects in the validation cohort. 3A) Global cognition is a Z‐score summarized composite score of 19 tests of different memory domains. The clinical consensus as to the presence of cognitive impairment at death accounts from an individual's baseline scores; thus, there is some overlap between groups in these scores. There was a significant main effect of cognition (*F*(1, 95) = 222.3, ****p < .0001) and pathology on global cognition score (*F*(1, 95) = 20.74, ****p < .0001). 3B) The mini mental state examination (MMSE) is a commonly used 30 question tool for relatively rapid assessment of cognitive impairment. There was a significant main effect of cognition (*F*(1, 95) = 114.8, ****p < .0001) and pathology (*F*(1, 95) = 8.331, **p = .0048) on MMSE, and a less significant interaction effect (*F*(1, 95) = 5.824, *p* = .0177). 3C) Global pathology score is a *Z*‐score summarized estimation of pathology load averaged across 5 brain regions, including those affected earlier in disease progression that the MT cortex. There is a significant main effect of pathology on global pathology score (*F*(1, 95) = 160.0, ****p < .0001) and a weakly significant interaction effect (*F*(1, 95) = 5.062, *p = .0268). There are no significant differences in 3D) age in years, 3E) education in years, and 3F) postmortem interval in hours

There were no significant differences in age, education, gender, or postmortem interval between groups (Figure 3). Group were divided based on their cognitive status (unimpaired vs. impaired) and AD pathology burden (Braak score; Figure 3, Table S3). As expected, there was a main effect of cognitive status on global cognition, a *Z*‐score normalized summary score of 19 cognitive tests across multiple cognitive domains, and minimental state examination (MMSE) score. For the purpose of this study, a Braak score of IV or below was considered low pathology, and V or above considered high, as this transition point is when the MT cortex begins to exhibit significant hyperphosphorylated tau pathology (Braak et al., 2006). As expected, there was a significant main effect of pathology on global pathology score.

### 3.5 Differential expression of *IGF2R* circRNA in MT cortex

**FIGURE 4:**
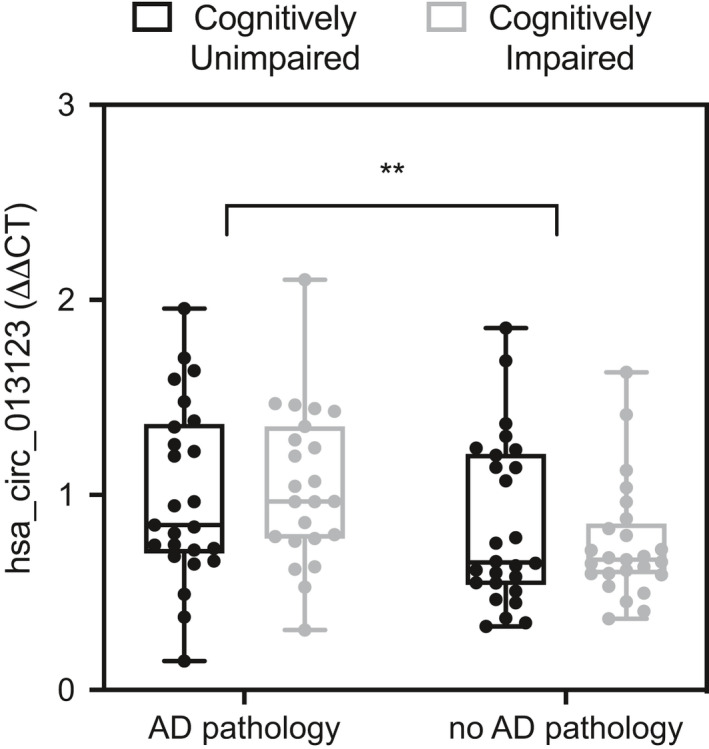
There is a main effect of AD pathology on hsa_circ_0131235 in MT cortex, *F*(1, 95) = 7.994, **p < .01

hsa_circ_0131235 was quantified in non‐RNaseR‐treated RNA from the validation cohort. RT‐qPCR was performed on 100 samples, and hsa_circ_0131235 was detected between 27 and 32 cycles. There was a significant main effect of pathology on hsa_circ_0131235 expression (*F*(1, 95) = 7.994). Mean hsa_circ_0131235 expression was increased in the MT cortex of individuals with high AD pathology relative to those with low AD pathology (Figure 4). No main effect of cognition or interactions between AD pathology and cognition was observed.

## 4 DISCUSSION

Noncoding RNAs have been shown to play a fundamental role in AD pathogenesis (Akhter, 2018), with dysregulated expression profiles of circRNA, microRNA, and mRNA identified in AD rat model hippocampi (Wang et al., 2018). A recent study published in Nature Neuroscience found that 31 circRNAs were significantly associated with Clinical Dementia Rating (CDR), nine with Braak Score, and nine with AD versus control status. Strikingly, circRNA expression accounted for more variation in CDR performance than ApoE4 status (Dube et al., 2019). hsa_circ_0131235 was not highlighted as significant in this sequencing based study, but it was not well powered to address the AD versus control pathology contrast. In our study, we confirmed hsa_circ_0131235 circularity and showed increased expression in the MT cortex in individuals with high AD pathology. As we did not find a significant correlation with cognitive ability, hsa_circ_0131235 may have potential as a marker of AD pathology burden. It is also important to note that while we did not find clear evidence of circularity post‐RNaseR digestion from the other three targets selected, this does not necessarily discount their being circular; there are reports of circRNAs that are degraded due to the presence of nicks in the sequence. Alternative approaches such as northern blotting and RNase H may be used to address this limitation of our study (Barrett & Salzman, 2016).

The “microRNA sponge” hypothesis of circRNA function suggests they inactivate corresponding microRNAs, indirectly upregulating protein expression (Lukiw, 2013; Piwecka et al., 2017). We can hypothesize a similar relationship between hsa_circ_0131235 and I*GF2R*. I*GF2R* facilitates Aß peptide clearance (Mellott et al., 2014), and expression levels have been correlated with altered functioning of lysosomal enzymes (Kar et al., 2006). Increased hsa_circ_0131235 may be part of a biological mechanism to prevent damage from Aß aggregation. IGF2/I*GF2R* signaling has been implicated in metabolic regulation and diabetes risk (Cianfarani, 2012) with circulating I*GF2R* associated with type two diabetes mellitus (T2DM) (Chanprasertyothin et al., 2015). Given diabetes is one of the strongest risk factors for the development of AD and a common comorbidity, dysregulation of IGF2 signaling through I*GF2R* may contribute to the neuropathology of AD.

CircRNA dynamic expression is generally distinct from linear isoforms of the same gene (Hansen et al., 2013; Piwecka et al., 2017). While we did not quantify I*GF2R* linear transcript expression in MT cortex in this experiment, RNA sequencing of over 500 samples from the same ROSMAP cohort shows no differences in linear IGFR2 mRNA expression between low and high Braak stage tissue from the transcriptionally similar dorsolateral prefrontal cortex (Bihlmeyer et al., 2019; Jager et al., 2018; Kang et al., 2011). It may therefore be likely that hsa_circ_0131235 is independently regulated to the linear transcript, but our current findings cannot give us information about potential mechanisms linking the two.

As our findings are limited to the middle temporal cortex, further studies quantifying *IGF2R* circRNA in additional cortical regions, such as the parietal cortex are necessary to evaluate its use as a biomarker for AD and to fully understand the mechanisms of hsa_circ_0131235 in AD pathogenesis. It would also be of benefit to assess the localization of hsa_circ_0131235 with regard to both cell types and subcellular domains using in situ hybridization or advanced versions of these techniques such as RNAscope (Wang et al., 2012). This would begin to address whether there is direct physical involvement of this circRNA with plaque structures or whether the mechanism of association may be more indirect. Additional investigations into *IGF2R* circRNA expression in other neurodegenerative pathologies are warranted to determine the disease specificity of this finding, as well as their potential for diagnosis and therapeutics.

## DISCLOSURES

All authors report no disclosures.

## AUTHOR CONTRIBUTION

I. Bigarré, Y. Guo, and B.C. Carlyle designed the experiments. I. Bigarré and B.A. Trombetta performed the experiments. I. Bigarré, B.A. Trombetta, and B.C. Carlyle analyzed the experiments. I. Bigarré, B.A. Trombetta, S.E. Arnold, and B.C. Carlyle wrote the manuscript. S.E. Arnold funded the work.

### PEER REVIEW

The peer review history for this article is available at https://publons.com/publon/10.1002/brb3.2048.

## Supporting information

Table S1

Table S2

Table S3

## Data Availability

The data that support the findings of this study are available from the corresponding author upon request.
